# Association between waist-to-height ratio and insulin resistance in patients with polycystic ovary syndrome: a meta-analysis

**DOI:** 10.3389/fendo.2025.1567787

**Published:** 2025-04-03

**Authors:** Yuxiang Lan, Chaohao Zhong, Dujin Li, Haobin Chen, Xu Dai

**Affiliations:** ^1^ Foshan Clinical Medical School, Guangzhou University of Chinese Medicine, Foshan, Guangdong, China; ^2^ The First Clinical College, Zunyi Medical University, Zunyi, Guizhou, China; ^3^ The First Clinical College, Guangdong Medical University, Zhanjiang, Guangdong, China

**Keywords:** waist-to-height ratio, polycystic ovary syndrome, WHtR, PCOS, insulin resistance, meta-analysis

## Abstract

**Background:**

Insulin resistance (IR) is regarded as a significant role in the pathophysiology of Polycystic Ovary Syndrome (PCOS), hence early identification of IR in PCOS patients is crucial for their treatment. Recent researches indicate that Waist-to-Height Ratio (WHtR) differs in patients with PCOS accompanied by IR compared to those with PCOS alone; however, a consensus has yet to be reached. This research presents a meta-analysis of current data to propose a straightforward new index for diagnosing PCOS with insulin resistance.

**Objective:**

This study aims to assess the correlation between WHtR and insulin resistance in individuals with polycystic ovarian syndrome by meta-analysis and the synthesis of research information. This study will offer prognostic insights into the onset of insulin resistance in individuals with polycystic ovaries.

**Methods:**

This systematic review has finalized registration (CRD42025638798) on the PROSPERO platform. We conducted a search of prominent databases, including PubMed, Embase, Web of Science, The Cochrane Library, and performed manual searches. Observational studies of relevance (cohort, case-control, or cross-sectional) published before January 13, 2025, were considered. The methodological quality of case-control studies and cohort studies was evaluated using the NOS tool. Simultaneously, AHRQ criteria were employed to evaluate cross-sectional research. The study outcomes were reported as mean ± sd. The aggregated data was analyzed using StataMP17.0.

**Results:**

A meta-analysis encompassing nine studies with 3012 participants identified a significant correlation between an increased WHtR and the onset of insulin resistance in individuals with polycystic ovarian syndrome (SMD: 1.07, 95% CI: 0.81, 1.32, p = 0.000).

**Conclusion:**

Patients with PCOS and insulin resistance will exhibit a greater WHtR compared to those with PCOS without insulin resistance. Thus, the incidence of insulin resistance in patients with polycystic ovary syndrome can be anticipated by monitoring the WHtR.

**Systematic review registration:**

https://www.crd.york.ac.uk/PROSPERO/view/CRD42025638798, identifier CRD42025638798.

## 1 Introduction

Polycystic ovarian syndrome is a prevalent gynecological endocrine and reproductive illness, affecting 10 - 13% of women of reproductive age (1), and its pathophysiology is intricate, including several variables, with no clear treatment now available. Insulin resistance is regarded as a significant contributor to the onset of PCOS (2), with research indicating that 30% - 60% of PCOS patients exhibit obesity and IR (3, 4). Furthermore, the majority of contemporary therapeutic treatment protocols for PCOS emphasize the enhancement of patients’ IR (3). Consequently, the prompt identification of insulin resistance in a patient is critically important for the diagnosis and management of polycystic ovary syndrome.

The prevailing gold standard for insulin resistance detection, the high insulin-glucose clamp test, is intricate and expensive (5). Other methodologies and indices for assessing abnormalities in glucose tolerance metabolism, such as the glucose tolerance test (GTT) and homeostasis model assessment of insulin resistance (HOMA-IR), entail significant operational and measurement demands, rendering them unsuitable for screening and early diagnosis. Consequently, there is a need to identify a straightforward and easily measurable index for diagnosing PCOS with insulin resistance. Consequently, it is essential to identify a straightforward and easily quantifiable index for the diagnosis of PCOS with insulin resistance. Considering that a majority of patients with PCOS and IR exhibit a pronounced inclination towards obesity, and recognizing the robust correlation between WHtR and both obesity and IR, a study on non-alcoholic fatty liver disease (NAFLD) revealed a reduction in patients’ body mass index (BMI) concomitant with a decrease in WHtR (6). This shows the importance of WHtR in assessing obesity and insulin resistance in individuals. In comparison to the existing high insulin-glucose clamp experiment, together with GTT and HOMA-IR, the assessment of WHtR is more straightforward and broadly applicable.

Consequently, we conducted a meta-analysis and review to explore the correlation between WHtR and IR in PCOS and its clinical implications for diagnosing IR.

## 2 Methods

### 2.1 Protocol

This study adhered to the most updated Preferred Reporting Items for Systematic Reviews and Meta-Analyses (PRISMA-2020) criteria for reporting (**Supplementary Table S1**). This systematic review was registered with PROSPERO (CRD42025638798) before initiation.

### 2.2 Study search

#### 2.2.1 Databases

Relevant observational studies were identified through PubMed, Embase, Web of Science, The Cochrane Library databases, and literature tracking methods such as hand searches.

#### 2.2.2 Search strategy

The search keywords included: “polycystic ovary syndrome,” “PCOS,” “insulin resistance,” “waist-to-height ratio,” “WHtR,” etc. Keywords were amalgamated utilizing Boolean logic, with disparate categories linked by “AND” and identical categories connected by “OR,” while searches incorporated combinations of subject and free terms. All articles published before January 13, 2025, were incorporated in the search. Detailed search methodologies and formulae for each database are available in **Supplementary Table S2**.

### 2.3 Study selection

#### 2.3.1 Inclusion criteria

(1) The study population comprised patients with PCOS (2); The study analyzed the disparities in WHtR levels between IR and non-insulin-resistant (non-IR) individuals (3); The study documented WHtR levels for the IR and Non-IR groups separately, expressed as mean ± sd (4); The study design was observational, including cohort, case-control, and cross-sectional studies.

#### 2.3.2 Exclusion criteria

(1) Non-thesis research, including reviews and conference abstracts (2); Animal research (3); In cases of duplication, only the entry with the most comprehensive information was retained.

### 2.4 Data extraction

Both investigators independently conducted the literature screening process in accordance with the aforementioned inclusion and exclusion criteria. Firstly, two reviewers independently read the titles and abstracts of the searched studies to exclude irrelevant studies. Then, the full text of the remaining studies was to be read and make the final selection. The inter-rater agreement for selecting studies with the Kappa score was calculated by IBM SPSS Statistics 25. Following the identification of papers for inclusion in the analysis, data extraction was conducted independently. The gathered information comprised the name and publication year of the first author, the study’s geographical region, details regarding the study population (age and sample size), the study design, diagnostic criteria for PCOS, the definition of insulin resistance, and the conclusion of the study (data for WHtR).

### 2.5 Quality assessment

The methodological quality of case-control and cohort studies was assessed using the NOS (Newcastle-Ottawa Scale) rating scale. The assessment encompassed three dimensions: subject selection, comparability, and exposure, comprising a total of 8 score criteria out of a possible 9 points. The assessment criteria were as follows: (1) A score of 7-9 was classified as a high-quality study; (2) a score of 4-6 was classified as a moderate-quality study; and (3) a score of less than 4 was classified as a low-quality study. The Agency for Healthcare Research and Quality (AHRQ) was employed to assess the quality of the cross-sectional studies, comprising 11 items with responses of “yes,” “no,” and “unclear,” while “yes” was assigned a score of 1, and “no” and “unclear” received a score of 0. The grading criteria were delineated as follows: (1) a score of 8-11 was classified as a high-quality study; (2) a score of 4-7 was classified as medium-quality; and (3) a score of 0-3 was classified as a low-quality study.

### 2.6 Statistical analysis

This study employed StataMP17.0 for the comprehensive analysis of data. The outcome indicator was a continuous variable, and the disparity in WHtR levels between the IR and non-IR groups was assessed for significance by gathering mean ± sd in both groups, with SMD (95% CI) serving as the effect size. The significance criterion was established at 0.05. Chi-square tests and Cochrane Q-tests were employed to assess heterogeneity among groups, whereas I² was utilized to quantify the extent of heterogeneity across the included studies. If I^²^ ≤ 50%, this indicated good homogeneity among trials, which were examined using a fixed-effects model. If I^²^ > 50%, it indicated inadequate homogeneity and elevated heterogeneity, necessitating analysis through a random effects model. When heterogeneity was excessive, the origin of heterogeneity was investigated by sensitivity analysis and subgroup analysis. We performed a sensitivity analysis by excluding literature one by one and analyzing the remaining included literature in general. The results of the sensitivity analysis are presented in the form of a sensitivity map. Egger’s test was employed to identify publication bias in the chosen studies. The Funnel plot test was not performed due to the small number of literatures included in the change study and the results presented by the Funnel plot test did not fully reflect the bias between publications.

## 3 Results

### 3.1 Included studies

**Figure 1:**
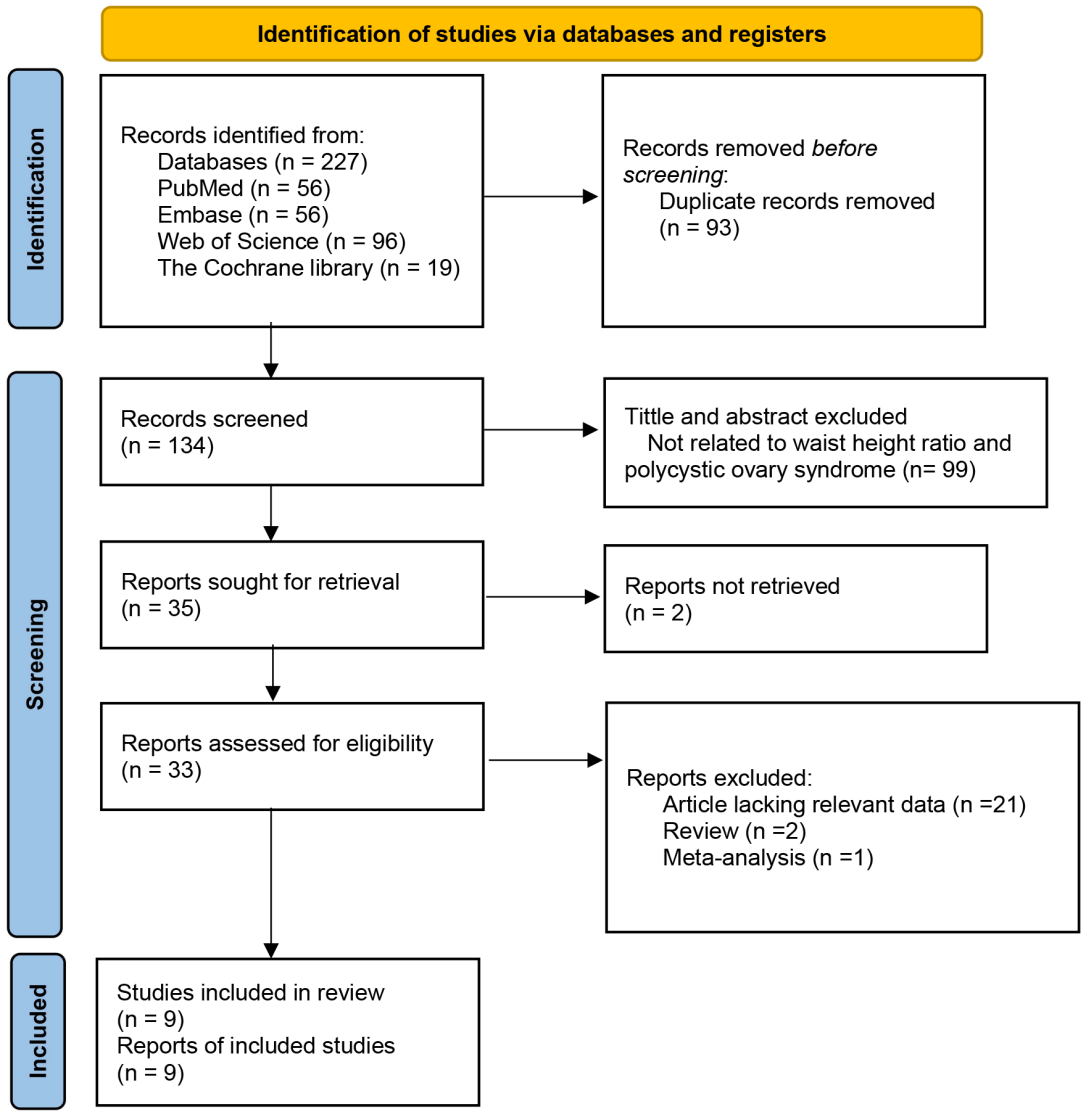
Contextual details of studies.

A total of 134 articles were acquired via four databases and manual searches. Following the evaluation of titles and abstracts pertinent to this study, 99 publications were removed, resulting in a total of 35 papers remaining with a Kappa score of 0.847. Two studies were inaccessible, and following a meticulous full-text examination of the remaining 33 papers, the application of inclusion and exclusion criteria, along with a thorough assessment, resulted in the exclusion of two reviews, one meta-analysis, and 21 articles without pertinent data. Ultimately, nine observational studies remained with an inter-rater agreement Kappa score of 0.807. The detailed information about the Kappa score between the reviewers was shown in **Supplementary Table S4**. A total of nine publications were included, as illustrated in **Figure 1**.

### 3.2 General characteristics of studies

Eight of the nine documents are in English, while one is in Portuguese. Seven cross-sectional studies and two case-control studies were incorporated. The experimental group consisted of 1721 individuals, while the control group contained 1291, resulting in a total of 3012 participants (**Table 1**).

**Table 1:** Contextual details of studies.

Title	First author, year	Country	Kind of research	Sample population and follow up time	Assessment of PCOS and insulin resistance stander
Wrist circumference: A new marker for insulin resistance in African women with polycystic ovary syndrome	Amisi et al. (7), 2020	Congo	prospective case-control study	The research have143 patients from Congo, which obtain 72 PCOS patients and 71 controls and it collect from October 2015 to December 2016.	The diagnostic criteria of PCOS in this research are 2003 Rotterdam criteria and the diagnostic criteria of IR is HOMA-IR.
Waist-to-height ratio and BMI as predictive markers for insulin resistance in women with PCOS in Kolkata, India	Bhattacharya et al. (8), 2021	India	cross-sectional study	The research have 66 PCOS patients from Calcutta, which collect form November 2018 to April 2019.	The diagnostic criteria of PCOS in this research are 2003 Rotterdam criteria and the diagnostic criteria of IR is HOMA-IR.
Lipid Profile in Relation To Anthropometric Indices And Insulin Resistance In Overweight Women With Polycystic Ovary Syndrome	Boshku et al. (9), 2019	Iran	cross-sectional study	The research have 63 PCOS patients from Gynecology and Endocrinology Outpatient Clinics of Tabriz University of Medical Sciences, Iran, which collect form January 2011 to August 2012.	The diagnostic criteria of PCOS in this research are 2022 Rotterdam criteria and the diagnostic criteria of IR is HOMA-IR, If patients HOMA-IR ≥3.8, It’s judge as IR.
Characteristics of Different Obesity Metabolic Indexes and their Correlation with Insulin Resistance in Patients with Polycystic Ovary Syndrome	Feng et al. (10), 2024	China	cross-sectional study	The research has 140 subjects with PCOS and 133 control subjects form Tianjin Medical University General Hospital, which collected from January 2018 to December 2022.	The diagnostic criteria of PCOS in this research are 2003 Rotterdam criteria and the diagnostic criteria of IR is HOMA-IR, If patients HOMA-IR ≥2.69, It’s judge as IR.
Evaluation of insulin resistance and plasma levels for visfatin and resistin in obese and non-obese patients with polycystic ovary syndrome	Gul et al. (11), 2015	Turkey	case-control study	The research has 37 patients with PCOS patients and 18 healthy volunteer	The diagnostic criteria of PCOS in this research are 2003 Rotterdam criteria
Optimal Cutoff Points for Anthropometric Variables to Predict Insulin Resistance in Polycystic Ovary Syndrome	Hatami et al. (12), 2017	Iran	cross-sectional study	224 PCOS patients	The diagnostic criteria of PCOS in this research are 2003 Rotterdam criteria and the diagnostic criteria of IR is HOMA-IR, If patients HOMA-IR ≥2.6, It’s judge as IR.
Lipid ratios and obesity indices are effective predictors of metabolic syndrome in women with polycystic ovary syndrome	Kałużna et al. (13), 2022	Poland	cross-sectional study	The research have 404 patients with PCOS and 192 healthy from the Department of Endocrinology of the Heliodor S' wie,cicki Clinical Hospital whoch was collect from September 2016 to March 2020.	The diagnostic criteria of PCOS in this research are 2003 Rotterdam criteria and the diagnostic criteria of MS are International Diabetes Federation (IDF) and the American Heart Association/National Heart, Lung, and Blood Institute (IDF-AHA/NHLBI) criteria (2009)
Anthropometric indices to predict insulin resistance in women with polycystic ovary syndrome in China	Liu et al. (2), 2019	China	cross-sectional study	The research have 1124 Patients with PCOS from the Reproductive Endocrinology Division of West China Second University Hospital of Sichuan University, Chengdu Sichuan, China, which collect from December 2011 and December 2016.	The diagnostic criteria of PCOS in this research are 2003 Rotterdam criteria and the diagnostic criteria of IR is HOMA-IR, If patients HOMA-IR ≥2.77, It’s judge as IR.
The waist-to-height ratio is a good predictor for insulin resistance in women with polycystic ovary syndrome	Zhu et al. (5), 2024	China	cross-sectional study	The research have 882 patients with PCOS from the Polycystic Ovary Syndrome Acupuncture plus Clomiphene Trial (PCOS Act), which was a large-sample, randomized controlled trial in China.	The diagnostic criteria of PCOS in this research are 2004 Rotterdam criteria and the diagnostic criteria of IR is HOMA-IR, If patients HOMA-IR ≥2.6, It’s judge as IR.

### 3.3 Quality assessment

This systematic review comprised nine publications. This review identified two cohort studies as high-quality, each with an NOS score of 9. Of the remaining seven cross-sectional studies, AHRQ scores ranged from 6 to 8; one study was classified as high quality, while six were deemed moderate quality. The overall quality was sufficient, as demonstrated in **Tables 2A** and **2B**.

**Table 2A:** NOS quality assessment results.

Author	a	b	c	d	e	f	g	h	Total scores
Amisi et al. (7), 2020	1	1	1	1	2	1	1	1	9
Gul et al. (11), 2015	1	1	1	1	2	1	1	1	9

a Representativeness of the exposed cohort.

b Selection of the non-exposed cohort.

c Ascertainment of exposure.

d Demonstration that outcome of interest was not present at start of study.

e Comparability of cohorts on the basis of the design or analysis.

f Assessment of outcome.

g Was follow-up long enough for outcomes to occur.

h Adequacy of follow up of cohorts.

**Table 2B:** AHRQ quality assessment results.

Author	1^a^	2^b^	3^c^	4^d^	5^e^	6^f^	7^g^	8^h^	9^i^	10^j^	11^k^	Total score
Bhattacharya et al. (8), 2021	1	1	1	1	1	1	1	1	0	0	0	7
Boshku et al. (9), 2019	1	1	1	1	1	1	1	1	0	0	0	8
Feng et al. (10), 2024	1	1	1	1	1	1	1	1	0	0	0	7
Hatami et al. (12), 2017	1	1	0	1	1	1	1	1	0	0	0	7
Kaluzna et al. (13), 2022	1	1	0	1	1	1	1	1	0	0	0	7
Liu et al. (2), 2019	1	1	0	1	1	1	1	0	0	0	0	6
Zhu et al. (5), 2024	1	1	0	1	1	1	1	1	0	0	0	7

a Define the source of information (survey, record review).

b List inclusion and exclusion criteria for exposed and unexposed subjects (cases and controls) or refer to previous publications.

c Indicate time period used for identifying patients.

d Indicate whether or not subjects were consecutive if not population-based.

e Indicate if evaluators of subjective components of study were masked to other aspects of the status of the participants.

f Describe any assessments undertaken for quality assurance purposes (e.g., test/retest of primary outcome measurements).

g Explain any patient exclusions from analysis.

h Describe how confounding was assessed and/or controlled.

i If applicable, explain how missing data were handled in the analysis.

j Summarize patient response rates and completeness of data collection.

k Clarify what follow-up, if any, was expected and the percentage of patients for which incomplete data or follow-up was obtained.

### 3.4 Outcome measures

**Figure 2:**
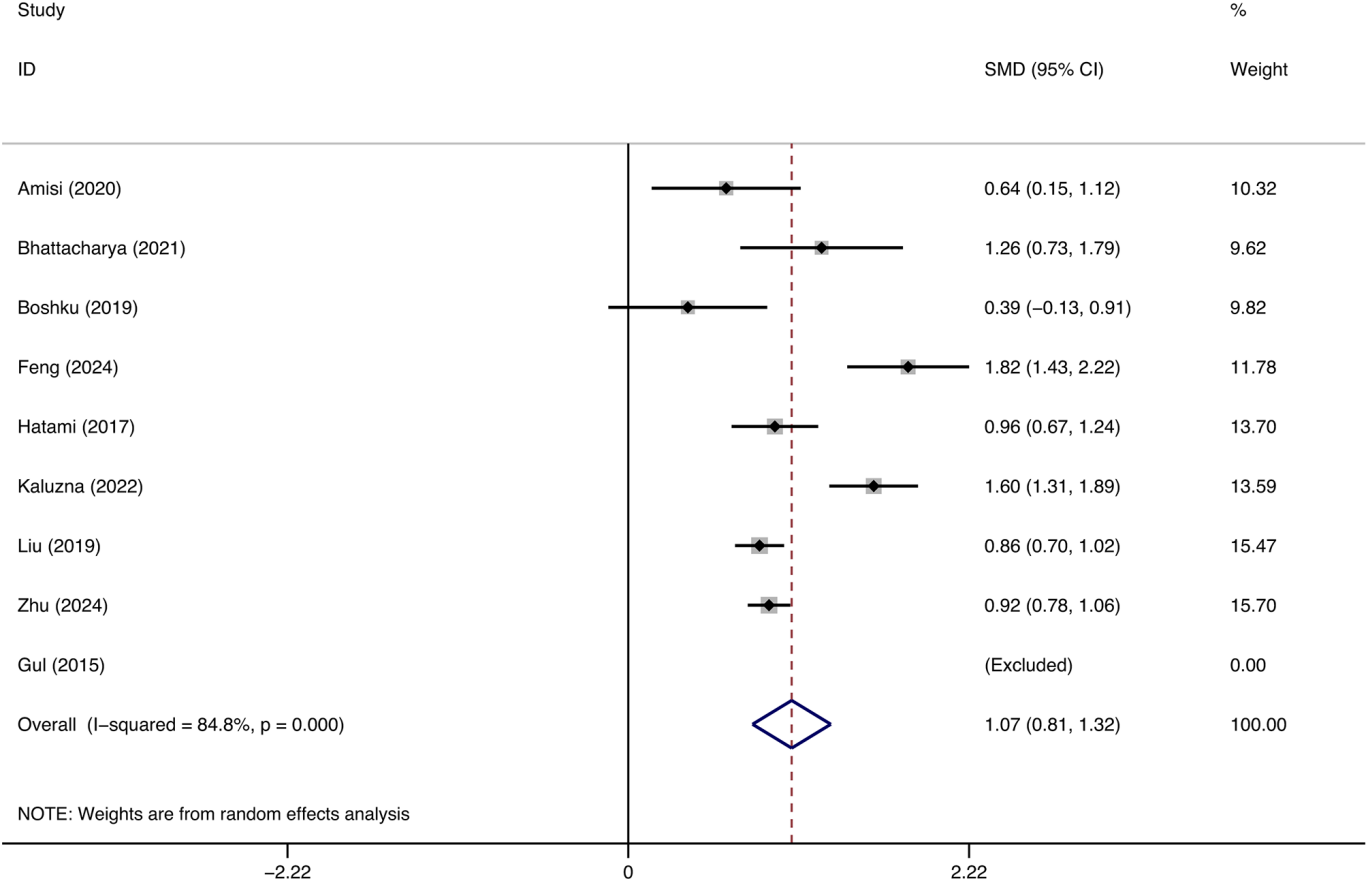
The association between waist-to-height ratio and insulin resistance in patients with polycystic ovary syndrome.

**Figure 3:**
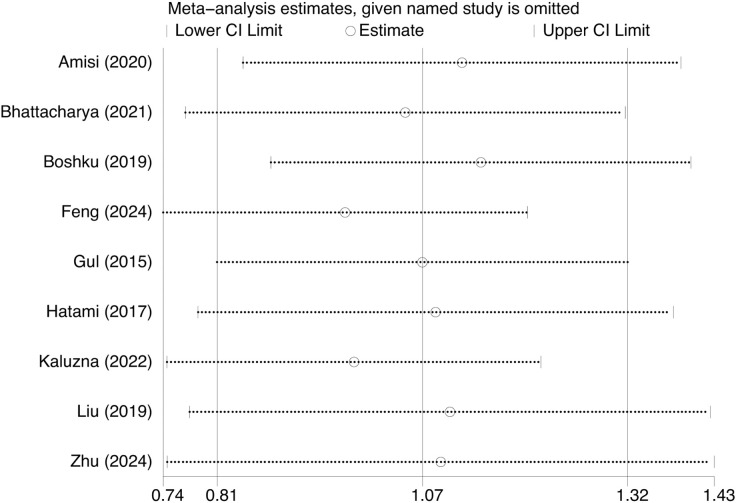
The sensitivity analyses of waist-to-height ratio.

This research comprised nine studies with 3012 patients diagnosed with PCOS to evaluate the correlation between WHtR and IR in this population. Patients with PCOS exhibiting IR demonstrated markedly elevated WHtR compared to those without IR (SMD: 1.07, 95% CI: 0.81, 1.32, p = 0.000, I^2^ = 84.8%) (**Figure 2**). Moreover, sensitivity analysis demonstrated that the aggregated results from this study were dependable (**Figure 3**). This reveals a notable correlation between an increased WHtR and the onset of IR in individuals with PCOS.

### 3.5 Subgroup analyses

**Figure 4:**
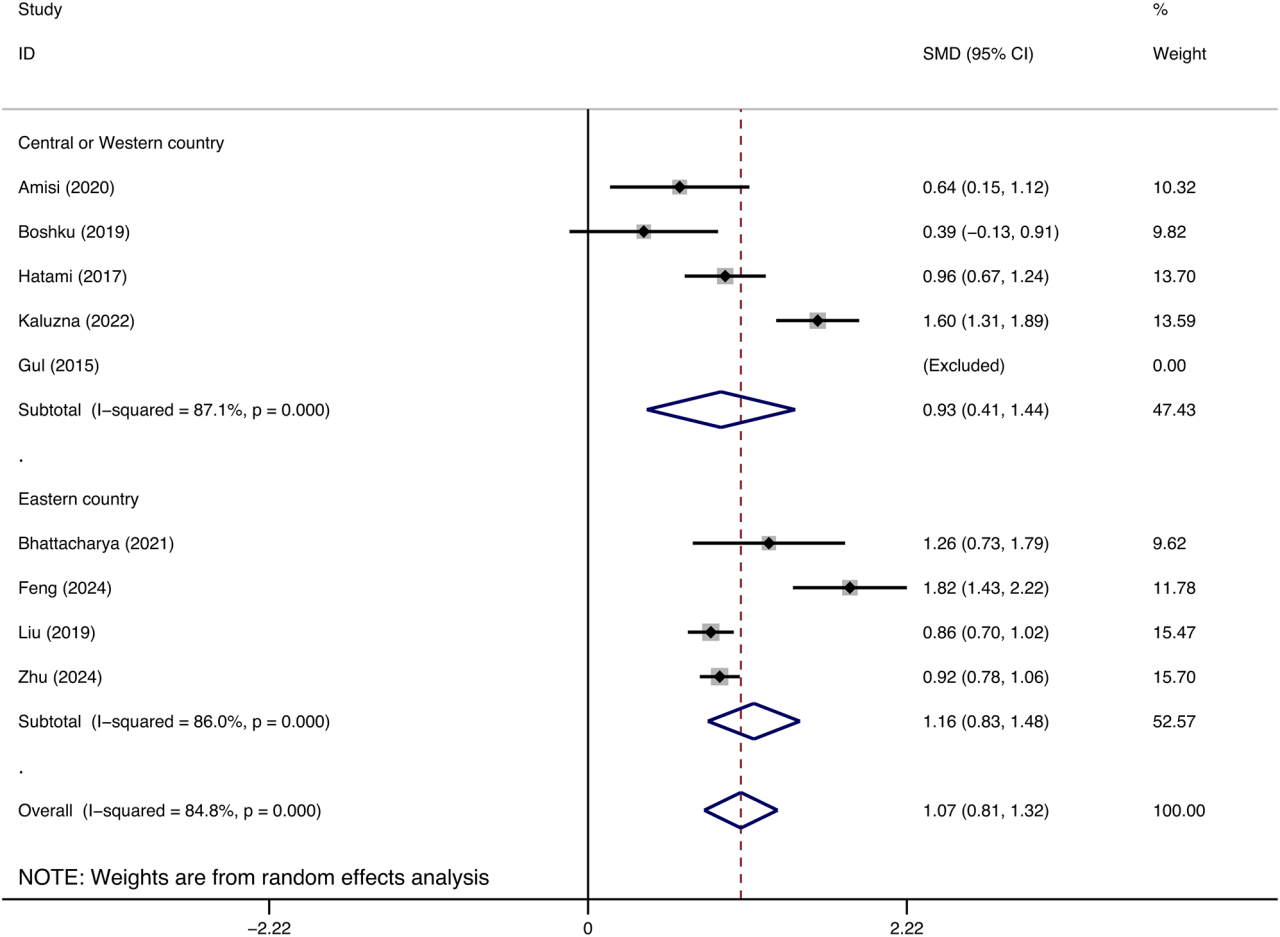
Subgroup analyses on difference of waist-to-height ratio and insulin resistance in patients with polycystic ovary syndrome in different areas.

The study was segmented into two subgroups according to geographic regions, with each analyzed individually. China and India were classified as Eastern nations, whereas Iran, Congo, and European nations were designated as Western and Central countries. The subgroup of Eastern countries included four studies demonstrating a significant correlation between increased WHtR and the onset of insulin resistance in individuals with polycystic ovarian syndrome (SMD: 1.16, 95% CI: 0.83, 1.48, p = 0.000, I^2^ = 86.0%). In a subgroup analysis of five studies from Western and Central nations, the WHtR of patients with polycystic ovarian syndrome exhibiting insulin resistance was markedly elevated compared to those without insulin resistance (SMD: 0.93, 95% CI: 0.41, 1.44, p = 0.000, I^2^ = 87.1%) (**Figure 4**).

### 3.6 Publication bias

Egger’s regression test indicated no substantial publication bias in the data (p > 0.05), as demonstrated in **Supplementary Table S3**.

## 4 Discussion

PCOS is an endocrine disorder associated with insulin resistance, hyperinsulinemia, dyslipidemia, obesity, and various metabolic alterations (11). The pathogenesis is intricate, with current understanding indicating a strong correlation between PCOS and insulin resistance (4). Most patients with insulin resistance exhibit disturbances in energy metabolism, resulting in alterations in physical test indicators, such as the prevalence of MASLD (Metabolic dysfunction-associated steatotic liver disease), which is linked to WHtR (14). In individuals with PCOS and insulin resistance, there are associated alterations in WHtR; nevertheless, the discrepancies in WHtR between those with PCOS and insulin resistance versus those with PCOS alone remain contentious and have yet to be standardized (2, 7, 8, 10, 15). After our meta-analysis, the results showed that there are some variations in WHtR between patients with PCOS alone and PCOS with IR, and the WHtR of patients with PCOS with IR is higher than that of patients with PCOS alone. The most common method for detecting insulin resistance in PCOS patients is the HOMA-IR index; however, this index is complex to measure and not easy to clinically screen for PCOS patients with IR. While BMI, a commonly used measure of patient body composition, is easy to measure, a global pooled study analysis showed that individuals may have uneven fat and muscle distribution due to a variety of factors, which may be overlooked by BMI when detecting obesity levels in patients (16). Meanwhile, the 2023 edition of the American Association of Clinical Endocrinologists and the American Endocrine Society’s Comprehensive Clinical Practice Guidelines for the Medical Care of Obese Patients in the U.S. also suggested that waist circumference can be used as an indicator of a patient’s risk of obesity, and that, in addition to BMI, waist circumference in different regions and populations should also be included as an indicator for the consideration of obesity (17). Therefore, the use of WHtR in the assessment of central obesity is more accurate than other anthropometric measures such as BMI (18), suggesting that WHtR seems to be able to be used in the pre-screening and prediction of patients with PCOS with IR. Although WHtR is more straightforward to measure and exhibits a distinction between patients with simple PCOS and those with PCOS accompanied by insulin resistance, several studies indicate that WHtR is significantly associated with central obesity. Its accuracy in evaluating central obesity surpasses that of other anthropometric measures, such as BMI (18), implying that WHtR may be effectively utilized for the pre-screening of patients with PCOS and IR. The existing studies also support this view, in a study from Kolkata, India, it was clearly demonstrated that WHtR can be used to predict IR in patients with PCOS in this region, and the critical value of WHtR in patients with PCOS with IR in this region was 0.62 (8), and in a study from Zhengzhou, China, it was demonstrated that WHtR is a good predictor for patients with POCS with IR in this region, and the critical value of WHtR in this region was 0.519 (5), and there are also studies from Iran and Poland also proved this view (13, 19). Although existing studies have proved that WHtR is able to play a role in the prediction of POCS with IR, the prediction cutoff values in the above studies only represent the situation in this region, and they still have limitations in the application of a large range of relevant indexes. Predictive cutoff values still need to be measured in further large-scale studies.

To further investigate the correlation between WHtR and PCOS in insulin-resistant patients, we performed a subgroup analysis based on the patients’ geographical regions. The findings indicated that the association between increased WHtR and PCOS with IR was more pronounced in Eastern countries, potentially linked to obesity resulting from Eastern dietary practices (20). Research indicates that patients with PCOS and IR are more prone to obesity (21), and individuals with obesity typically exhibit elevated WHtR (22). Consequently, it can be inferred that energy metabolism disorders in PCOS patients with IR, resulting in obesity, significantly contribute to increased WHtR values.

Nonetheless, our study possesses certain limitations, including significant heterogeneity. The discrepancy may be attributable to the limited sample size of the study participants. Furthermore, there is a paucity of research regarding the correlation between insulin resistance and WHtR in polycystic ovarian syndrome, and the limited number of studies may have resulted in analytical bias. The analysis also failed to account for the impact of varying patient disease duration on WHtR, hence introducing potential bias in the results. We also found that the HOMA-IR, an indicator of insulin resistance, was not consistent in some of the studies, which ranged from 2.6-3.8, and most of the studies had a cut-off value of 3 or less for the HOMA-IR, with just one study having a cut-off value of 3 or more, so our calculated correlation of WHtR may be biased. Moreover, our study included mainly articles from cross-sectional studies, so although statistically there is a strong correlation between WHtR and PCOS with IR, the causal relationship between the two remains to be explored in further studies.

Given that enhancing insulin resistance in individuals with polycystic ovary syndrome by exercise remains the primary therapeutic approach (3, 4), identifying a straightforward and easily measurable indication for screening polycystic ovary syndrome with insulin resistance is crucial. Patients diagnosed with PCOS in the early stages can have their WHtR measured, and if it exceeds the normal threshold, diet and exercise interventions can be initiated in advance, and further testing of glucose metabolism indicators can be performed for diagnosis, which can help in the treatment and prognosis of the disease. However, even though WHtR is easy to measure and strongly correlated with PCOS with IR, patients with suspected PCOS with IR need to be tested for glucose to confirm the diagnosis according to the Rotterdam Guidelines 2023 (1), and therefore we believe that WHtR can be used as a pre-screening aid, which is important in underdeveloped areas where glucose is not detected. In the meantime, more relevant studies should be conducted in the future, for example, by implementing prospective studies to examine whether changes in WHtR are associated with changes in insulin resistance over time in patients with PCOS.

In conclusion, a significant link exists between WHtR and PCOS with IR. It holds significant clinical relevance for the future prediction of PCOS onset with insulin resistance and for screening PCOS patients for preexisting insulin resistance.

## 5 Conclusion

In conclusion, this meta-analysis established a robust correlation between WHtR and polycystic ovary syndrome with insulin resistance, offering significant insights into the utilization of WHtR for the pre-diagnosis and screening of PCOS with IR. Nevertheless, additional epidemiological and experimental investigations are required to ascertain the clinical applicability of WHtR for diagnosing insulin resistance in individuals with PCOS.

## Data Availability

The datasets presented in this study can be found in online repositories. The names of the repository/repositories and accession number(s) can be found in the article/**Supplementary Material**.

## Supplementary material

Supplementary Table 1 PRISMA 2020 checklist.

Supplementary Table 2 Search strategies of each database.

Supplementary Table 3 The Egger’s regression test of air pollution.

Supplementary Table 4 The detailed information about the Kappa score between the reviewers.
