# Supplementary material for: Association between waist-to-height ratio and insulin resistance in patients with polycystic ovary syndrome: a meta-analysis
Source: Front Endocrinol (Lausanne). 2025 Apr 3;16:1567787. doi: 10.3389/fendo.2025.1567787 (PMC12003136; doi:10.3389/fendo.2025.1567787)
Supplement: Supplementary Table 2 — Search strategies of each database. [file Table2.docx]

The retrieval time: 20250113

**Sup Table 1 PubMed (n = 56)**

| Search | Query |
| --- | --- |
| #1 | "polycystic ovary syndrome"[MeSH Terms] OR "polycystic ovary syndrome"[All Fields] OR "polycystic ovarian syndrome"[All Fields] OR PCOS[All Fields] |
| #2 | "insulin resistance"[MeSH Terms] OR "insulin resistance"[All Fields] |
| #3 | "waist height ratio"[MeSH Terms] OR ("waist height"[All Fields] AND "ratio"[All Fields]) OR "waist height ratio"[All Fields] OR ("waist"[All Fields] AND "height"[All Fields] AND "ratio"[All Fields]) OR "waist to height ratio"[All Fields] OR "WHtR"[All Fields] |
| #4 | #1 AND #2 AND #3 |

**Sup Table 2 Embase (n = 56)**

| Search | Query |
| --- | --- |
| #1 | ('polycystic ovary syndrome'/exp OR 'polycystic ovary syndrome' OR pcos OR 'polycystic ovarian syndrome') |
| #2 | ('insulin resistance'/exp OR 'insulin resistance') |
| #3 | ('waist-to-height ratio'/exp OR 'waist-to-height ratio' OR whtr OR 'waist height ratio'/exp OR 'waist height ratio' OR 'waist to height ratio'/exp OR 'waist to height ratio') |
| #4 | #1 AND #2 AND #3 |

**Sup Table 3 Web of Science (n = 96)**

| Search | Query |
| --- | --- |
| #1 | (polycystic ovary syndrome) OR PCOS OR (polycystic ovarian syndrome) (All Fields) |
| #2 | **insulin resistance** (All Fields) |
| #3 | (waist-to-height ratio) OR WHtR OR (waist height ratio) OR (waist to height ratio) (All Fields) |
| #4 | #1 AND #2 AND #3 |

**Sup Table 4 The Cochrane library (n = 19)**

| Search | Query |
| --- | --- |
| #1 | MeSH descriptor: [Polycystic Ovary Syndrome] explode all trees |
| #2 | ((polycystic ovary syndrome) OR PCOS OR (polycystic ovarian syndrome)):ti,ab,kw (Word variations have been searched) |
| #3 | #1 OR #2 |
| #4 | MeSH descriptor: [Insulin Resistance] explode all trees |
| #5 | (insulin resistance):ti,ab,kw (Word variations have been searched) |
| #6 | #4 OR #5 |
| #7 | MeSH descriptor: [Waist-Height Ratio] explode all trees |
| #8 | ((waist-to-height ratio) OR WHtR OR (waist height ratio) OR (waist to height ratio)):ti,ab,kw (Word variations have been searched) |
| #9 | #7 OR #8 |
| #10 | #3 AND #6 AND #9 |
