# Supplementary material for: Association between waist-to-height ratio and insulin resistance in patients with polycystic ovary syndrome: a meta-analysis
Source: Front Endocrinol (Lausanne). 2025 Apr 3;16:1567787. doi: 10.3389/fendo.2025.1567787 (PMC12003136; doi:10.3389/fendo.2025.1567787)
Supplement: Supplementary Table 3 — The Egger’s regression test of air pollution. [file Table3.docx]

**Excluding studies by reading the title and abstract**

| Reviewer1 | Reviewer2 | | | Total |
| --- | --- | --- | --- | --- |
|  | Exclude | Include | Unclear |  |
| Exclude | 90 | 3 | 4 | 97 |
| Include | 0 | 33 | 1 | 34 |
| Unclear | 1 | 0 | 2 | 3 |
| Total | 98 | 34 | 2 | 134 |

Kappa:0.847

**Excluding studies by reading the full text**

| Reviewer1 | Reviewer2 | | | Total |
| --- | --- | --- | --- | --- |
|  | Exclude | Include | Unclear |  |
| Exclude | 21 | 0 | 2 | 23 |
| Include | 0 | 9 | 0 | 9 |
| Unclear | 0 | 1 | 0 | 1 |
| Total | 20 | 11 | 2 | 33 |

Kappa:0.807
