# Supplementary material for: Association between waist-to-height ratio and insulin resistance in patients with polycystic ovary syndrome: a meta-analysis
Source: Front Endocrinol (Lausanne). 2025 Apr 3;16:1567787. doi: 10.3389/fendo.2025.1567787 (PMC12003136; doi:10.3389/fendo.2025.1567787)
Supplement: Supplementary Table 4 — The detailed information about the Kappa score between the reviewers. [file DataSheet1.docx]

Egger's test

----------------------------------------------------------------------------

> --

Std_Eff | Coefficient Std. err. t P>|t| [95% conf. interva

> l]

-------------+--------------------------------------------------------------

> --

slope | .8364106 .2508815 3.33 0.016 .2225256 1.4502

> 96

bias | 1.417483 2.008476 0.71 0.507 -3.49708 6.3320

> 46

----------------------------------------------------------------------------

> --
